# Case Report: Heterogeneous endoscopic and histological presentations of gastric and gastroesophageal metastases from breast carcinoma: a three-case series from a Malaysian tertiary center

**DOI:** 10.3389/fonc.2026.1933062

**Published:** 2026-10-05

**Authors:** Guo Hou Loo, Yin Ping Wong, Nur Fa’izah Ab Muin, Kean Leong Koay, Mariyam Nahuza, Shahrun Niza Abdullah Suhaimi, Nik Ritza Kosai

**Affiliations:** 1 Upper Gastrointestinal, Bariatric and Metabolic Surgery Unit, Department of Surgery, Faculty of Medicine, Universiti Kebangsaan Malaysia (Hospital Canselor Tuanku Muhriz), Kuala Lumpur, Malaysia; 2 Department of Pathology, Faculty of Medicine, Universiti Kebangsaan Malaysia (Hospital Canselor Tuanku Muhriz), Kuala Lumpur, Malaysia; 3 Department of Radiotherapy and Oncology, Faculty of Medicine, Universiti Kebangsaan Malaysia (Hospital Canselor Tuanku Muhriz), Kuala Lumpur, Malaysia; 4 Breast and Endocrine Surgical Unit, Department of Surgery, Faculty of Medicine, Universiti Kebangsaan Malaysia (Hospital Canselor Tuanku Muhriz), Kuala Lumpur, Malaysia

**Keywords:** breast carcinoma, E-cadherin, gastric metastasis, gastroesophageal junction, HER2-low, invasive lobular carcinoma, linitis plastica, receptor discordance

## Abstract

**Background:**

Gastric metastasis from breast carcinoma is uncommon and is most often associated with invasive lobular carcinoma (ILC), although carcinoma of no special type (NST) and mixed tumors also occur. Metastatic deposits closely mimic diffuse-type gastric adenocarcinoma, and the distinction from a primary gastric tumor carries major therapeutic consequences. Estrogen receptor (ER), progesterone receptor (PR), and human epidermal growth factor receptor 2 (HER2) status may differ between the primary tumor and its metastasis, with direct implications for systemic therapy.

**Case descriptions:**

We report three women managed at a single Malaysian tertiary center. Case 1 (56 years old; metachronous interval of approximately 4 years) had an ER-positive, HER2 3+ (immunohistochemistry [IHC]) carcinoma of no special type that presented as a circumferential Siewert III gastroesophageal junction (GOJ) tumor with gastric satellites; the metastasis retained E-cadherin and was HER2 2+ with a non-amplified *in situ* hybridization (ISH) result, a discordance from the HER2 3+ primary. Case 2 (47 years old; synchronous) presented with a linitis plastica-like pattern and gastric outlet obstruction (GOO) from an ER-positive, HER2-negative carcinoma; the primary showed patchy E-cadherin and the metastasis showed focal E-cadherin, consistent with a mixed ductal-lobular phenotype; PR expression was lost between the primary (strong, >90%) and the metastasis (negative). Case 3 (91 years old; effectively synchronous, approximately 6-year course) had a lobular carcinoma with E-cadherin loss and a decline in PR expression (from focal weak positivity in the primary to negative in the metastasis), with a protracted course dominated by recurrent feeding-tube complications. In each case, the diagnosis rested on morphology, a breast-oriented IHC panel, and comparison with the known breast primary rather than on any single marker.

**Conclusion:**

This series illustrates that gastric and GOJ metastases from breast carcinoma are morphologically and endoscopically heterogeneous and are not confined to E-cadherin-negative ILC. E-cadherin reflects tumor phenotype and cell cohesion rather than the organ of origin, and it should not be used to distinguish a breast metastasis from a primary gastric carcinoma. Because clinically relevant ER, PR, and HER2 discordance may occur, these biomarkers should be reassessed in metastatic tissue, with results interpreted proportionately to the available evidence.

## Introduction

Breast carcinoma is the most common malignancy in women worldwide; however, metastasis to the stomach is rare. Clinical and autopsy series indicate that the incidence of gastric metastasis from breast cancer is approximately 0.2%–0.7% clinically and up to approximately 18% at autopsy, with most reported cases attributed to invasive lobular carcinoma (ILC) (1–3). Relative to invasive carcinoma of no special type, ILC displays a particular tropism for the peritoneum, gastrointestinal tract, gynecological organs, and leptomeninges, whereas pulmonary metastases are comparatively less frequent (4–6). This phenomenon is clinically important out of proportion to its frequency because metastatic deposits in the stomach or gastroesophageal junction (GOJ) closely simulate diffuse-type gastric adenocarcinoma, with discohesive, single-file, cord-like, or infiltrative growth, and the two entities demand fundamentally different management. A primary gastric cancer may be considered for resection, whereas a gastric metastasis from breast cancer is treated with systemic therapy (7, 8).

Two issues merit emphasis. First, although gastric metastasis is most often lobular, carcinoma of no special type and mixed phenotypes also metastasize to the stomach, so the metastasis need not display lobular morphology. E-cadherin is helpful for breast histological phenotyping but does not indicate the organ of origin: its loss or aberrant expression is also well recognized in diffuse-type gastric adenocarcinoma (9), and retained E-cadherin does not exclude a breast metastasis. Second, the ER, PR, and HER2 profile of a metastasis may differ from that of the primary tumor. Such discordance is clinically consequential: loss of ER or PR may reduce the expected benefit of endocrine therapy, while a change in HER2 status can alter eligibility for HER2-directed agents. In particular, the demonstration of low-level HER2 expression (HER2-low) (10) now confers eligibility for trastuzumab deruxtecan, which improved progression-free and overall survival in HER2-low advanced breast cancer in the DESTINY-Breast04 trial (11), with consistent benefit in an Asian subgroup (12).

We present three women with gastric and/or GOJ metastases from breast carcinoma managed at a single Malaysian tertiary referral center. Together, they span the temporal range from synchronous to late metachronous disease and a spectrum of endoscopic, morphological, and receptor findings. We use them to illustrate the diagnostic pitfalls of this presentation, the appropriate and cautious use of immunohistochemistry, and the value of reassessing receptor status in metastatic tissue.

## Case descriptions

The key clinicopathological features of the three cases are summarized in **Table 1**, while their clinical timelines, including the timing of gastric or gastroesophageal metastasis and major treatment events, are illustrated in **Figure 1**.

**Table 1:** Clinicopathological summary of the three cases.

Feature	Case 1	Case 2	Case 3
Age / ethnicity	56, Malay	47, Malay	91, Chinese
Primary histology	NST (reported ductal); E-cadherin not tested on primary	NST; E-cadherin patchy (aberrant) → mixed ductal-lobular	Suggestive of lobular differentiation
Timing of gastric spread	Metachronous (~4 years)	Synchronous (~months)	Synchronous at diagnosis (~6-year course)
Presentation	Asymptomatic; CT-detected gastric thickening	Dysphagia, weight loss, GOO	Dysphagia; surveillance-detected
Endoscopic pattern	Circumferential Siewert III GOJ tumor + gastric satellites	Linitis plastica-like; pyloric stenosis	Thickened mucosa, lesser curvature body–antrum
Site	GOJ + gastric body	Diffuse (cardia–pylorus)	Antrum / lesser curvature
Key IHC	GATA3+, CK7+, CK20−, E-cadherin retained	CKAE1/AE3+, GATA3+, E-cadherin focal	CK7+, GATA3+, CK20−, E-cadherin −
ER / PR (metastasis)	ER 80% / PR <5%	ER+ / PR− (primary: ER/PR >90%)	ER strong (primary) / PR Allred 0 (primary: focal weak +)
HER2 (primary → metastasis)	3+ IHC (no ISH) → 2+ IHC, ISH ratio 1.24 (HER2-low)	Negative → negative	Negative → 1+
Receptor discordance	HER2 category (3+ IHC → HER2-low)	PR (strong + → negative)	PR (focal weak + → negative)
Systemic therapy	Trastuzumab + letrozole; T-DXd considered	Paclitaxel → fulvestrant + goserelin	Letrozole → tamoxifen (sequential)
Supportive care	Surveillance; NGJ/stent if progresses	NGJ feeding; ascites drainage	Recurrent NGJ reinsertion; UGIB

CT, computed tomography; ER, estrogen receptor; GOJ, gastroesophageal junction; GOO, gastric outlet obstruction; HER2, human epidermal growth factor receptor 2; IHC, immunohistochemistry; ISH, in-situ hybridization; NGJ, naso-gastrojejunal; NST, no special type; PR, progesterone receptor; T-DXd, trastuzumab deruxtecan; UGIB, upper gastrointestinal bleeding.

**Figure 1:**
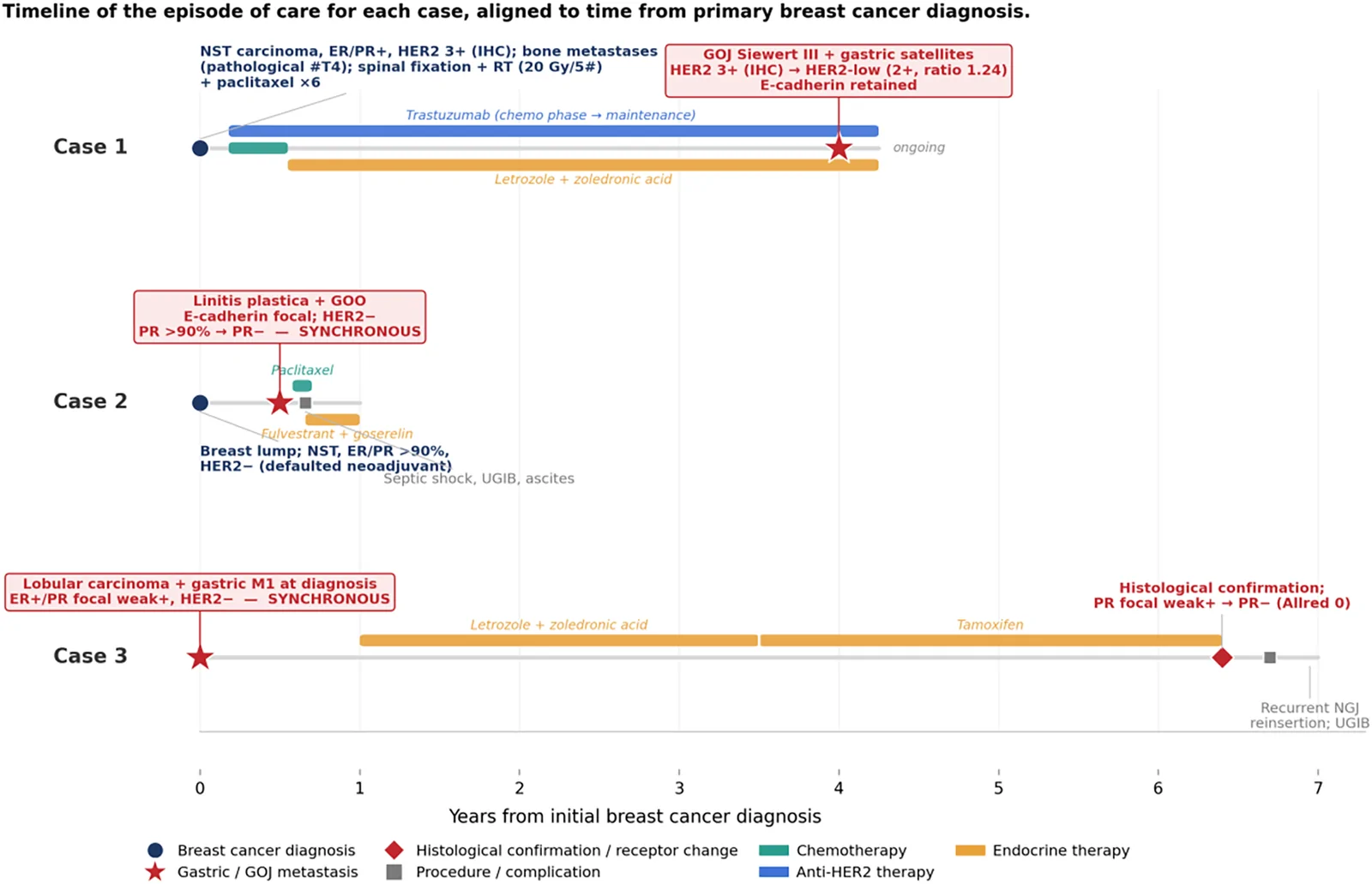
Timeline of the episode of care for each case, aligned to the time from the initial breast cancer diagnosis. Treatment durations are shown as bars (anti-HER2, chemotherapy, and endocrine therapy); discrete clinical events are shown as markers. The alignment illustrates the temporal spectrum: gastric or gastroesophageal metastasis was metachronous in Case 1 (approximately 4 years) but effectively synchronous in Cases 2 and 3. Callouts highlight key findings, including the HER2 3+ to HER2-low discordance (Case 1) and the loss or decline of PR expression in the metastases of Cases 2 and 3. GOJ, gastroesophageal junction; GOO, gastric outlet obstruction; ILC, invasive lobular carcinoma; NGJ, nasogastrojejunal; RT, radiotherapy; UGIB, upper gastrointestinal bleeding.

### Case 1: metachronous, HER2-discordant gastroesophageal junction metastasis of no-special-type carcinoma

A 56-year-old Malay woman was diagnosed in 2022 with ER-positive, PR-positive, HER2 3+ (IHC) right breast carcinoma. At initial presentation, she had back pain and lower-limb weakness secondary to a pathological T4 vertebral fracture; magnetic resonance imaging confirmed multiple bone metastases, and staging computed tomography (CT) showed a spiculated right breast mass infiltrating the skin and pectoralis with bilateral axillary nodes but no visceral metastases. The breast core biopsy (performed at another center) was reported as infiltrating ductal carcinoma. She underwent spinal instrumentation and T4 fixation; the vertebral biopsy showed metastatic carcinoma with a gland-forming pattern that was CK7-positive, ER-positive, and PR-negative, and was reported as consistent with a breast origin. She received palliative radiotherapy to C7–T6 (20 Gy in five fractions) and six cycles of paclitaxel with trastuzumab, followed by maintenance trastuzumab, letrozole, and zoledronic acid every three months. The breast mass and bone deposits remained stable on serial CT, and left ventricular ejection fraction was maintained at 61%–63% on surveillance echocardiography during trastuzumab therapy.

Approximately 4 years after the initial diagnosis, surveillance CT showed new gastric wall thickening with minimal ascites, prompting upper gastrointestinal endoscopy (OGDS). This revealed a circumferential Siewert III GOJ tumor with small satellite tumors along the greater curvature (**Figure 2A**). Biopsies of the GOJ and gastric body showed malignant cells in discohesive sheets, some forming tubular structures, with intracytoplasmic mucin. The tumor cells were positive for GATA3 and CK7 and negative for CK20, with retained membranous E-cadherin; ER was positive (80%, moderate to strong), PR was weak (<5%), and Ki-67 was 25%. HER2 was 2+ by IHC, and dual-color ISH gave a HER2/chromosome-17 ratio of 1.24 (not amplified), corresponding to a HER2-low metastasis. This differs from the HER2 3+ (IHC) primary, on which ISH had not been performed (**Figures 3A–F**). The morphology, the breast-oriented panel, and comparison with the known breast primary supported a breast origin; a primary GOJ adenocarcinoma was considered less likely given the concurrent breast history, the receptor profile, and the retained low-level ER expression. The gastric metastasis and the earlier bone deposit shared the same estrogen receptor-positive, CK7-positive, PR-negative immunophenotype, both expressed GATA3, and both were reported as consistent with a breast origin (**Figures 3K–N**); the more gland-forming appearance at the bone site and the more discohesive pattern in the stomach reflect recognized cross-site morphological variation of breast metastases rather than two separate primary tumors. At the time of reporting, she remained free of dysphagia (weight 55 kg, hemoglobin 11.1 g/dL). Because she was asymptomatic with stable disease, systemic therapy was continued unchanged (trastuzumab and letrozole); the HER2-low result was noted as making trastuzumab deruxtecan an option should the disease progress, and rechallenge chemotherapy was also considered for the future.

**Figure 2:**
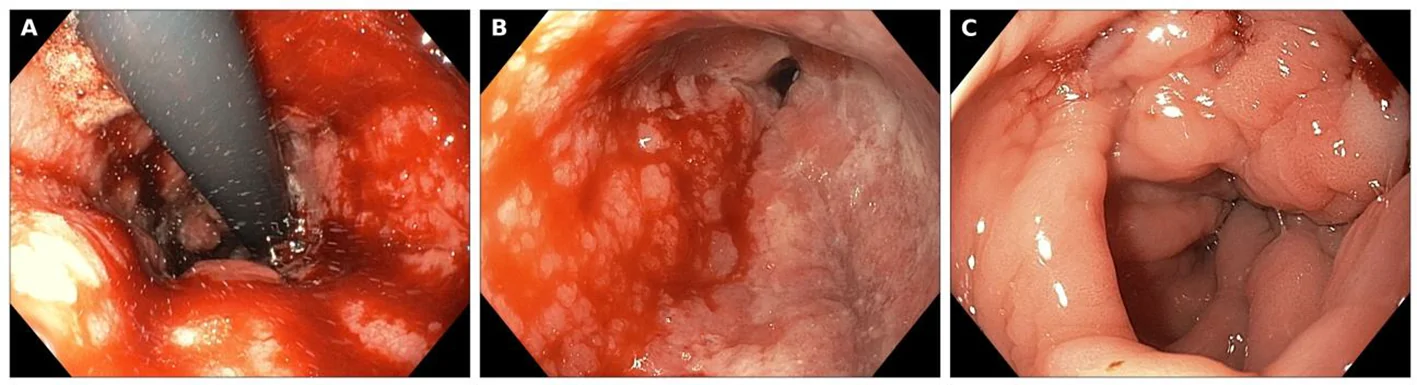
Representative endoscopic appearances, illustrating three distinct patterns that mimic primary gastric disease. **(A)** Case 1: circumferential Siewert III gastroesophageal junction tumor with friable, nodular mucosa. **(B)** Case 2: diffusely thickened, non-distensible, infiltrated gastric mucosa with a linitis plastica-like appearance. **(C)** Case 3: subtle thickening of the lesser-curvature gastric mucosa detected on surveillance endoscopy. Images are de-identified endoscopic stills; panel letters are shown at the upper left of each panel.

**Figure 3:**
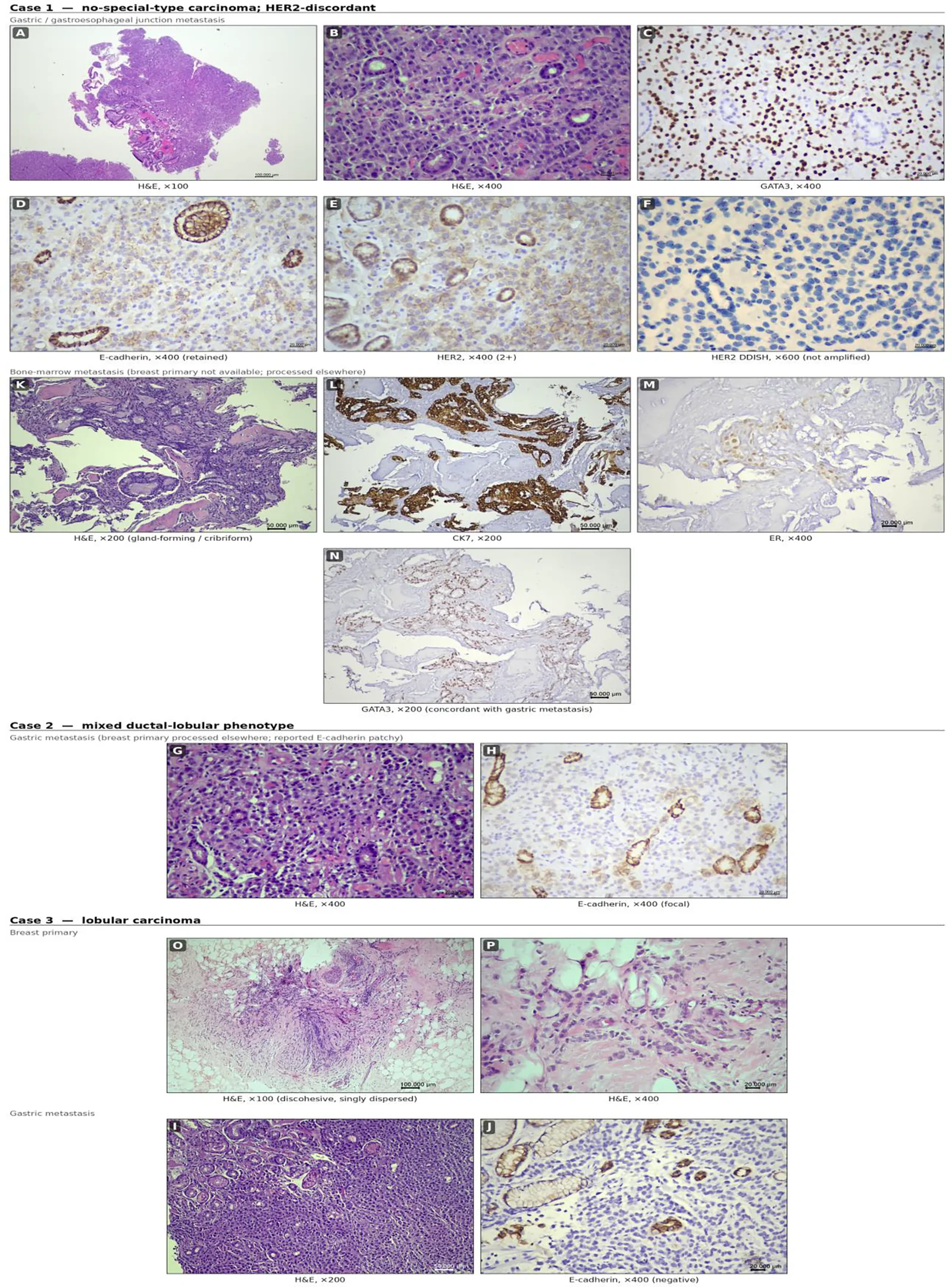
Paired and metastatic histopathology. For each case, the gastric metastasis is shown with additional available breast or non-gastric metastatic tissue to allow direct morphological and immunophenotypic comparison. Case 1, gastroesophageal junction metastasis **(A–F)**: **(A)** low-power biopsy (H&E, ×100); **(B)** infiltration by malignant cells in short cords and trabeculae (H&E, ×400); **(C)** diffuse nuclear GATA3 (×400); **(D)** retained membranous E-cadherin (×400); **(E)** HER2 2+ by immunohistochemistry (×400); and **(F)** dual-color dual-hapten *in situ* hybridization (DDISH) showing no HER2 amplification (×600). Case 1, bone-marrow metastasis **(K–N)**: **(K)** malignant cells in complex, focally cribriform glands within marrow spaces (H&E, ×200); **(L)** diffuse CK7 (×200); **(M)** estrogen receptor (×400); and **(N)** diffuse nuclear GATA3 (×200), concordant with the GATA3 expression of the gastric metastasis and confirming a shared breast lineage; the breast primary was processed at another center and was not available for imaging. Case 2, gastric metastasis **(G–H)**: **(G)** infiltration by loosely cohesive sheets of malignant cells (H&E, ×400) with **(H)** focal E-cadherin (×400); the breast primary was processed elsewhere, and its reported E-cadherin was patchy. Case 3, breast primary **(O, P)**: **(O)** infiltration by loosely cohesive, singly dispersed cells (H&E, ×100) and **(P)** mildly pleomorphic cells with vesicular nuclei and eosinophilic cytoplasm (H&E, ×400). Case 3, gastric metastasis **(I–J)**: **(I)** discohesive malignant cells (H&E, ×200) with **(J)** E-cadherin negativity (×400). Scale bars are as provided by the imaging system, magnifications are as stated, and panel letters appear at the upper left of each panel.

### Case 2: synchronous linitis plastica-like gastric metastasis with outlet obstruction

A 47-year-old Malay woman presented with a right breast lump. Core biopsy (performed at another center) confirmed invasive carcinoma of no special type, Bloom–Richardson grade 2, with strong ER and PR expression (>90%) and negative HER2 (score 0); a subsequent supplementary stain on the primary showed patchy membranous E-cadherin of weak to moderate intensity, indicating an aberrant, non-uniform pattern rather than the strong, diffuse membranous expression of typical ductal carcinoma. Staging CT showed no distant metastases but an incidental multinodular goiter with retrosternal extension. She was counseled for neoadjuvant therapy but defaulted owing to concerns about chemotherapy. Within months, she developed progressive dysphagia to solids with regurgitation of partially digested food and substantial weight loss (approximately 20 kg over four months).

OGDS showed thickened, edematous gastric mucosa with an inability to distend the stomach and a linitis plastica-like appearance; the pylorus could not be intubated. Random gastric biopsies showed neoplastic cells in discohesive small aggregates and single-file arrangements infiltrating the lamina propria. The tumor cells were positive for CKAE1/AE3, GATA3, and ER and negative for PR and HER2, with focal E-cadherin expression (**Figures 3G, H**); the concordance between the patchy E-cadherin of the primary and the focal E-cadherin of the metastasis supports a mixed ductal-lobular phenotype. Whereas the breast primary had shown strong PR expression (>90%), the gastric metastasis was PR-negative, indicating a loss of PR expression between the primary and the metastasis. Taken together with the recent ER-positive, HER2-negative breast primary and the hormone receptor–positive profile of the gastric lesion, the appearances were reported as metastatic carcinoma of breast origin; a primary diffuse gastric carcinoma was considered less likely on clinical and immunophenotypic grounds. Repeat emergency endoscopy for gastric outlet obstruction (GOO) confirmed a circumferential tumor extending from cardia to pylorus with pyloric stenosis traversable only by a slim scope; a nasogastrojejunal (NGJ) feeding tube was placed under direct vision (**Figure 2B**). Her course was complicated by post-chemotherapy septic shock, upper gastrointestinal bleeding from the gastric metastasis, and malignant ascites requiring drainage. She received single-agent paclitaxel followed by fulvestrant and goserelin for symptom control and disease modification.

### Case 3: effectively synchronous lobular gastric metastasis with reduced PR expression

A 91-year-old Chinese woman, a nursing-home resident with hypertension, dyslipidemia, atrial fibrillation on anticoagulation, and a prior cerebrovascular accident, was diagnosed in 2019 with left breast invasive carcinoma reported as suggestive of lobular differentiation, with gastric involvement identified at presentation. The primary was ER-positive (strong, 90%), PR focally and weakly positive, and HER2 1+ (negative) (**Figures 3O, P**). Surgery was considered inappropriate given her age and comorbidities. She received sequential endocrine therapy, letrozole (with zoledronic acid) followed by tamoxifen, over several years, with eventual clinical progression.

Surveillance imaging showed diffuse gastric antral wall thickening with enhancing perigastric nodes. OGDS showed thickened gastric mucosa along the lesser curvature from the body to the antrum (**Figure 2C**). Antral biopsy showed malignant cells in discohesive sheets and cords within a desmoplastic stroma. The tumor cells were positive for CK7 and GATA3 and negative for CK20 and E-cadherin (**Figures 3I, J**). Interpreted together with the documented lobular breast primary and the clinical course, the findings were consistent with a lobular breast metastasis rather than a primary diffuse gastric carcinoma. On the metastasis, PR was negative (Allred 0), and HER2 was 1 +. Compared with the primary, in which PR had been only focally and weakly positive, this represents a decline from low-level to absent PR expression rather than loss of strong expression, and it accompanied clinical progression on endocrine therapy. Her subsequent course was dominated by the morbidity of malignant gastric infiltration: recurrent NGJ feeding-tube blockage and dislodgement requiring repeated endoscopic reinsertion over several months, an episode of upper gastrointestinal bleeding, and progressive functional decline (ECOG 3).

## Discussion

These three cases, considered together, offer several practical lessons about the recognition and characterization of gastric metastasis from breast carcinoma.

First, gastric metastases from breast carcinoma are heterogeneous and are not confined to E-cadherin-negative ILC. Across the series, the metastases ranged from a tumor of no special type with retained E-cadherin (Case 1), through a mixed ductal-lobular tumor with focal E-cadherin (Case 2), to a lobular tumor with E-cadherin loss (Case 3), the last concordant with the documented lobular primary. E-cadherin should be interpreted in relation to tumor phenotype and cell cohesion rather than as a marker of organ of origin. Its expression may be retained, reduced, heterogeneous, or lost in breast carcinoma, while aberrant or lost expression is also well recognized in diffuse-type gastric adenocarcinoma (9); retained expression therefore does not exclude a breast metastasis, and its loss does not establish one. In each of our cases the diagnosis rested on the combination of clinical history, morphology, comparison with the known breast primary, and a breast-oriented immunohistochemical panel, rather than on any single stain.

GATA3 supported breast lineage in each case, but it is not by itself decisive. GATA3 can be expressed in a proportion of primary gastric and gastroesophageal adenocarcinomas, and its sensitivity falls in triple-negative and higher-grade tumors, so it is best used within a panel rather than as a standalone marker. Where the distinction from a primary gastric tumor is genuinely in doubt, and if tissue permits, breast-lineage markers such as TRPS1, GCDFP-15, and mammaglobin, and gastrointestinal or gastric markers such as CDX2, HNF4A, CEA, and mucin stains can be added; TRPS1 in particular retains sensitivity in lobular and triple-negative tumors. These additional stains were not performed in the present cases, and we regard this as a limitation. In practice, the most decisive element was direct comparison with the known breast primary in the context of the clinical history.

Second, the cases illustrate the range of endoscopic and morphological mimicry. Case 1 presented as a circumferential Siewert III GOJ tumor indistinguishable at endoscopy from a primary junctional adenocarcinoma; Case 2 produced a linitis plastica-like appearance with gastric outlet obstruction; and Case 3 was detected as subtle mucosal thickening on surveillance. Each could plausibly have been approached as a primary upper gastrointestinal cancer, including consideration of resection, had the breast history and immunophenotype not redirected management toward systemic therapy. Rather than a novel recommendation, these cases reinforce established practice: in a patient with a history of breast cancer, a new gastric lesion should prompt consideration of metastatic disease and selection of an appropriate immunohistochemical panel with comparison to the primary (3, 6–8, 13, 14).

Third, and most important, ER, PR, and HER2 status may differ between the primary tumor and its metastasis, and reassessing these biomarkers in metastatic tissue can be clinically meaningful. In Case 1, HER2 changed from 3+ by IHC on the primary to 2+ with a non-amplified ISH result on the metastasis, corresponding to a HER2-low state; because ISH was not performed on the primary, the change is best described as a discordance in the HER2 category rather than proven loss of amplification. In Case 2, PR was strongly expressed in the primary (>90%) but negative in the gastric metastasis, a loss of PR expression. In Case 3, PR declined from focal weak positivity to negativity, a shift from low-level to absent expression rather than loss of strong positivity. Apparent discordance of this kind has several possible explanations that cannot be separated in individual cases: true biological evolution of the tumor, treatment-related clonal selection (including selection under prior trastuzumab and endocrine therapy), intratumoral and spatial heterogeneity sampled differently by small biopsies, and preanalytical or analytical differences between specimens, platforms, and scoring criteria over time. These caveats argue for cautious interpretation, but they do not remove the practical message: biomarkers should be repeated on metastatic tissue because the result can change management. Where a metastasis is reclassified as HER2-low, as in Case 1, trastuzumab deruxtecan becomes a potential option based on the DESTINY-Breast04 trial and its Asian subgroup (11, 12), although in our patient, who was asymptomatic with stable disease, the finding did not change immediate treatment, and access in middle-income settings remains constrained by cost. Receptor conversion between primary and metastatic breast cancer is well documented and underpins the recommendation to rebiopsy and reprofile metastatic deposits (15).

Finally, the temporal spectrum was wide, from effectively synchronous presentation (Cases 2, 3) to a metastasis emerging 4 years after the primary (Case 1). Gastric metastasis can therefore be an early, dominant manifestation or a late event during otherwise stable disease, and a change on surveillance imaging (as in Case 1) should prompt endoscopic evaluation even in an asymptomatic patient.

The strength of this report is the juxtaposition of three contrasting cases with endoscopic and histopathological documentation and, where available, a comparison with the breast primary or another metastatic site. A few data limitations are relevant to interpretation. None of the patients underwent breast surgery; therefore, pathological tumor size, nodal status, and definitive pathological stage of the primary tumors were not available. In addition, E-cadherin and HER2 were not reassessed in every primary tumor, limiting complete primary-metastatic tumor comparisons. Despite these constraints, the present cases illustrate the marked heterogeneity in the clinical, endoscopic, and histopathological presentation of gastric metastases from breast carcinoma.

## Conclusion

Gastric and gastroesophageal metastases from breast carcinoma are uncommon but clinically consequential, and they show heterogeneous endoscopic and histological appearances that mimic primary diffuse gastric cancer. E-cadherin expression reflects tumor phenotype rather than the primary site and should not be used to determine the origin of a gastric lesion. GATA3 is a useful supportive marker but should be interpreted within a broader clinicopathological and immunohistochemical panel and, wherever possible, alongside direct comparison with the breast primary. Because clinically relevant ER, PR, and HER2 discordance may occur, these biomarkers should be reassessed in metastatic tissue, with any treatment implications kept proportionate to the case-level evidence. A history of breast cancer should lower the threshold for biopsy and for considering metastatic disease when a new gastric lesion is encountered.

## Patient perspective

Case 1: After several years of stable disease, the patient reported feeling well and was surprised to learn that cancer had spread to the junction of her gullet and stomach. She was reassured that she had no difficulty swallowing and that further treatment options were available. She chose to continue her current therapy and regular follow-up.

Case 2: The patient described how progressive difficulty in swallowing and marked weight loss affected her daily life and her ability to eat with her family. She recalled being fearful of chemotherapy at first and felt that a clear explanation and support from the team helped her accept treatment directed at relieving her symptoms. She found that the feeding tube, although initially distressing, allowed her to maintain nutrition.

Case 3: The patient described the burden of repeated feeding-tube problems and expressed appreciation for the supportive care she received.

## Data Availability

The original contributions presented in the study are included in the article/supplementary material. Further inquiries can be directed to the corresponding author.
